# 晚期非小细胞肺癌探索性四线及以上治疗的回顾性分析

**DOI:** 10.3779/j.issn.1009-3419.2014.12.03

**Published:** 2014-12-20

**Authors:** 仙凤 王, 媚娟 黄, 莉 任, 泳 徐, 潞 李, 梅 侯, 瑾 王, 枫 彭, 江 朱, 永生 王, 铀 卢

**Affiliations:** 1 610041 成都，四川大学华西医院肿瘤中心胸部肿瘤科 Department of Thoracic Oncology, Cancer Center, West China Hospital, West China Medical School, Sichuan University, Chengdu 610041, China; 2 610041 成都，四川大学华西医院生物治疗国家重点实验室 State Key Laboratory of Biotherapy, West China Hospital, West China Medical School, Sichuan University, Chengdu 610041, China

**Keywords:** 肺肿瘤, 四线治疗, 生存及预后因素, Lung neoplasms, Fourth-line therapy, Survival and prognostic factors

## Abstract

**背景与目的:**

晚期非小细胞肺癌的一、二线治疗策略已达成广泛共识，对于三线治疗，最新的美国国立综合癌症网络（National Comprehensive Cancer Network, NCCN）指南中已有相关推荐。本研究旨在研究晚期非小细胞肺癌四线及以上治疗结果及影响因素。

**方法:**

回顾性分析我院肿瘤中心四线及以上治疗的140例晚期非小细胞肺癌患者的临床资料，对其有效率、生存及预后因素等进行分析。

**结果:**

18例（12.9%）部分缓解（partial response, PR)，36例（25.7%）疾病稳定（stable disease, SD），疾病控制率（disease control rate, DCR）为38.6%。中位总生存时间（overall survival, OS）及四线治疗后OS分别为31个月及10.1个月。四线治疗中位无疾病进展生存时间（progression free survival, PFS）为2.6个月。单因素及多因素分析均显示不同治疗选择及是否后续治疗是四线OS独立预后因素。未发现四线PFS的独立预后因素。化疗组较靶向组四线中位OS更长（11.7个月*vs* 7.1个月，*P*=0.013）。单药及双药化疗，首次及反复使用表皮生长因子受体络氨酸激酶抑制剂（epidermal growth factor receptor tyrosine kinase inhibitor, EGFR-TKI）四线中位OS无差异。

**结论:**

对有条件接受四线治疗的晚期非小细胞肺癌患者，接受四线治疗能获得生存的延长。四线治疗在晚期非小细胞肺癌的地位值得前瞻性临床试验进一步探讨。

## Abstract (en)

**Background and objective:**

A common consensus has been reached regarding first- and second-line therapies for advanced non-small cell lung cancer (NSCLC). The newest guideline from National Comprehensive Cancer Network (NCCN) also provides recommendations for third-line therapy. This study focused on fourth-line and enhanced treatments for advanced NSCLC. Treatment results and impact factors were analyzed and discussed.

**Methods:**

A total of 140 patients with advanced NSCLC were selected and their data were analyzed. Response rate, survival rate, and prognostic factors were evaluated.

**Results:**

Among the 140 patients, 12.9% (18 cases) showed partial response, 25.7% (36 cases) suffered from a stable disease, and 38.6% (54 cases) exhibited a disease control rate. Median overall survival (OS) and fourth-line therapy OS were 31 months and 10.1 months, respectively. The median progression free survival (PFS) of fourth-line therapy was 2.6 months. Univariate and multivariate analyses indicated different therapy regimens and suggested whether or not patients should undergo follow-up treatments. These parameters were independent prognostic factors of the OS of the fourth-line therapy; by contrast, no independent impact factor of PFS was found. Chemotherapy resulted in better median OS in fourth-line therapy than in targeted therapy (11.7 months *vs* 7.1 months, *P*=0.013). Considering the median OS of fourth-line therapy, we observed that single agent therapy did not significantly differ from double agent therapy; likewise, we found that first-time usage did not significantly differ from multiple usage of epidermal growth factor receptor tyrosine kinase inhibitor.

**Conclusion:**

Fourth-line therapy is recommended to increase the survival of advanced NSCLC patients. Nevertheless, the role of fourth-line therapy in advanced NSCLC should be further assessed in clinical trials.

晚期非小细胞肺癌的一、二线治疗策略已达成广泛共识，即根据基因状态、组织学类型、一般状况等因素选择方案。可应用的药物包括三代化疗药物、抗新生血管生成药物、表皮生长因子受体酪氨酸激酶抑制剂（epidermal growth factor receptor-tyrosine kinase inhibitor, EGFR-TKI）等。但中位无进展时间（progression free survival, PFS）均有限，大部分患者均在获益一年内进展^[1]^。对多项Ⅲ期临床研究的分析显示：约有30%-70%的患者接受了二线进展后的治疗^[2]^。对于三线治疗，最新的美国国立综合癌症网络（National Comprehensive Cancer Network, NCCN）指南中推荐使用培美曲塞（仅限于非鳞癌）、多西他赛、吉西他滨及厄洛替尼^[1]^。在临床实践中，部分患者在接受三线治疗后仍保留较好的一般状态及器官功能，有条件接受进一步治疗。同时，关于治疗意愿的调查研究^[3]^表明：多数患者并不在意治疗副反应，要求接受以延长生命为目的的积极抗肿瘤治疗。在一项纳入417例患者的回顾性研究^[4]^中，分别有54%、26%、10%、5%的患者接受了二、三、四、五线治疗。按照患者意愿进行相关治疗是否合理已经成为必需回答的问题。但目前国内外很少有相关研究结果报道。我们选择接受四线及以上治疗的晚期非小细胞肺癌患者进行回顾性分析，旨在探讨晚期非小细胞肺癌四线治疗的临床意义。

## 1 资料及方法

### 1.1 临床资料

选择2003年1月-2013年1月在四川大学华西医院肿瘤中心接受抗肿瘤治疗的Ⅳ期非小细胞肺癌患者共1, 791例，重新按照美国癌症联合会（American Joint Committee on Cancer, AJCC）分期7.0进行分期。共有140例患者接受四线或更多线治疗。通过查阅住院及门诊记录、影像学资料，回顾分析或调阅表皮生长因子受体（epidermal growth factor receptor, *EGFR*）等基因检测结果并随访等获得资料。包括：年龄、性别、婚姻状况（配偶生存状态）、吸烟史、一线及四线治疗体力状态（Eastern Cooperative Oncology Group, ECOG）评分、组织学、*EGFR*基因突变情况、各线治疗方案、疗效及生存数据等。

### 1.2 统计学方法

建立EXCEL数据库，采用SPSS 17.0进行相关分析。通过*Kaplan-Meier*方法评估生存，预后因素分析采用*Log-rank*检验及*Cox*回归分析。总生存时间（overall survival, OS）的定义为从初次诊断至死亡的时间或最后一次随访时间，四线治疗OS定义为开始四线治疗至死亡或最后一次随访时间。四线治疗疗效评价按照实体瘤疗效评价标准（Response Evaluation Criteria in Solid Tumors, RECIST）1.1进行。

## 2 结果

### 2.1 患者临床特征

87%的患者 < 65岁，88%患者初诊时的体力状态ECOG评分为0分-1分，而接受四线治疗时49%患者为2分及以上。83%患者病理类型为非鳞癌。75例（53%）患者能够检测*EGFR*突变，41例（29%）敏感突变阳性，34例（24%）阴性。仅2例检测到有意义的ALK重排（表 1）。

**1:** 患者临床特点（*n*=140） Patient characteristics (*n*=140)

Characteristics	No. of patients	Percent (%)
Age (yr)		
≥65	18	12.9
< 65	122	87.1
Median (range)	53（19-82）	
Gender		
Male	73	52
Female	67	48
Performance status (First-line)		
0-1	123	88
2	17	12
Performance status (Fourth-line)		
0-1	71	51
2-3	69	49
Smoking history		
Former	48	34
Never	92	66
Spouse		
Living	130	93
Sick, divorced, unmarried	10	7
Histology		
Squamous cell carcinoma	24	17
Others	116	83
*EGFR* mutation status		
Positive	41	29
Negative	34	24
Unknown	65	47
Interval between first- and fourth-line therapy		
≥12 mo	103	74
< 12 mo	37	26
Stage Ⅳ when diagnosed	140	100
Whether contain EGFR-TKI or not before fourth-line therapy		
Yes	106	76
No	34	24
EGFR-TKI: epidermal growth factor receptor tyrosine kinase inhibitor.		

### 2.2 治疗

接受四线和五线及更多治疗的患者分别为140/1791（7.8%）例及71/1, 791例（4.0%）。患者最多接受了九线治疗。仅有10例患者在整个过程中未接受小分子靶向药物的治疗，其中仅1例患者*EGFR*突变阳性。在四线治疗中，62.9%接受化疗，其中36.0%为双药化疗，共有49例患者接受含培美曲赛的化疗。33.6%口服EGFR-TKI单药。5例患者接受其他小分子靶向药物治疗。12.9%接受化疗联合靶向治疗。32例（22.9%）患者接受姑息放疗，其中22例全脑放疗或γ刀治疗，6例椎体放疗，3例肺部原发灶及1例锁骨上淋巴结放疗。患者前三线的化疗比例分别为73%、61%、51%，小分子靶向药物的比例分别为17%、34%、40%（表 2）。

**2:** 治疗情况 Treatment status

Treatment	First-line (*n*)	Second-line (*n*)	Third-line (*n*)	Fourth-line (*n*)
Chemotherapy	102	85	70	70
Single-agent	3	9	18	19
Double-agent	99	76	52	51
Combined therapy	14	8	12	18
Targeted therapy	24	47	58	52
EGFR-TKI	24	45	54	47
Others	0	2	4	5
Combined therapy: chemotherapy combined targeted therapy.				

### 2.3 疗效及生存

111例（79.3%）患者能评价四线治疗疗效，其中18例（12.9%）部分缓解（partial response, PR），36例（25.7%）稳定（stable disease, SD），疾病控制率（disease control rate, DCR）38.6%。化疗组8例（6.0%）PR，小分子靶向药物7例（5%）PR，联合治疗组3例（2%）PR。所有患者均能评价生存。随访至2013年9月，30例患者仍生存。四线治疗中位PFS为2.6个月，中位OS及四线治疗OS分别为31个月及10.1个月。

### 2.4 四线治疗预后因素

四线治疗PFS及OS的预后因素分析结果具体见表 3。性别、年龄、病理、不同*EGFR*基因状态等因素均不是四线PFS的独立预后因素，仅吸烟史在单因素分析中显示出统计学差异。四线接受化疗、靶向、化疗联合靶向治疗患者的PFS分别为：2.7个月、2.6个月、2.5个月，整体PFS无统计学差异（*P*=0.221）。单因素及多因素分析均显示：不同的治疗方案、四线治疗后的后续治疗是四线治疗OS的独立预后因素。化疗、靶向治疗及化疗联合靶向治疗组的中位四线治疗OS分别为11.7个月、7.1个月及8.7个月，总体差异有统计学意义（*P*=0.012）。两两比较显示：化疗组较靶向治疗组四线OS延长（*P*=0.013）（图 1）。化疗组较化疗联合靶向组有延长的趋势，但未显示出统计学差异（*P*=0.065）。靶向治疗组与联合治疗组无差异（*P*=0.951）。四线治疗后有、无后续治疗的四线中位OS分别为14.2个月及6.1个月，差异有统计学意义（*P* < 0.001）。不同ECOG评分、*EGFR*基因状态不是四线治疗OS的独立预后因素。

**3:** 预后分析结果 Results of prognostic analysis

	Fourth-line mPFS (mo)	Univariate (Multivariate)	Fourth-line mOS (mo)	Univariate (Multivariate)
Gender		0.211 (0.781)		0.216 (0.695)
Male	2.2		8.0	
Female	3.7		11.2	
Age		0.362 (0.155)		0.363 (0.049^△^)
≥65	3.2		16.7	
< 65	2.6		9.3	
Smoking history		0.027^△^ (0.156)		0.054 (0.237)
Yes	2.2		7.1	
No	3.5		11.0	
Histology		0.095 (0.160)		0.291 (0.458)
Squamous cell carcinoma	1.9		6.9	
Others	2.8		10.9	
*EGFR* mutation status		0.891 (0.676)		0.992 (0.591)
Positive	2.1		9.9	
Negative	2.6		10.8	
Unknown	3.0		9.3	
Interval between first- and fourth-line therapy		0.347 (0.502)		0.115 (0.238)
≥12 mo	2.8		11.4	
< 12 mo	2.0		8.2	
Performance status (Fourth-line)		0.661 (0.741)		0.381 (0.861)
0-1	2.8		11.0	
≥2	2.6		8.7	
Fourth-line therapy		0.221 (0.313)		0.012^△^ (0.007^△^)
Chemotherapy	2.7		11.7	
Targeted therapy	2.6		7.1	
Combined therapy	2.5		8.7	
Continued therapy after fourth-line therapy				
Yes			14.2	< 0.001^△^ (< 0.001^△^)
No			6.1	
mPFS: median progression free survival; mOS: median overall survival; ^△^*P* < 0.05.				

**1:**
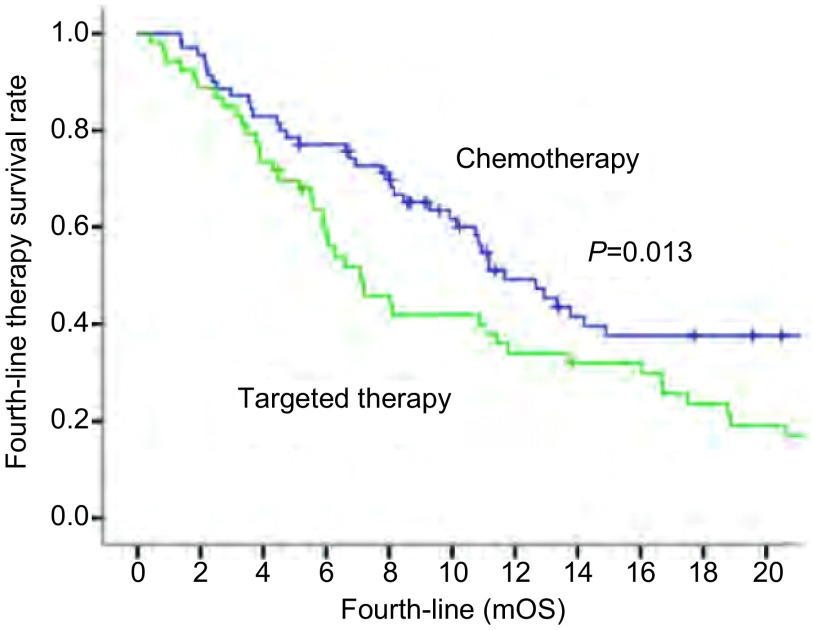
通过两两比较显示：化疗较靶向治疗四线OS延长（*P*=0.013） Pairwise comparison show that fourth-line OS is longer in chemotherapy than in targeted therapy (*P*=0.013)

### 2.5 亚组分析

我们分别对化疗组及靶向治疗组进行了关于OS的亚组分析。单药化疗与双药化疗组的四线治疗中位OS分别为11.2个月及12.7个月，无统计学差异（*P*=0.906）。化疗方案是否含有培美曲赛对四线治疗中位OS无影响（11.7个月及11.2个月，*P*=0.936）。在47例口服单药EGFR-TKI患者中，首次给予EGFR-TKI组四线治疗中位OS 8.0个月，再次给予组7.1个月（*P*=0.143）。*EGFR*突变状况不影响四线EGFR-TKI治疗的中位OS（*P*=0.351）。2例服用克唑替尼患者分别已服药15个月及5个月，目前均未进展。2例使用索拉菲尼患者均在2个月内死亡。1例凡德他尼患者四线治疗OS达12.6个月。四线治疗后有、无后续治疗对四线化疗及靶向治疗OS影响有统计学差异（22.0个月*vs* 7.8个月，*P*=0.004；11.4个月*vs* 3.9个月，*P*=0.013），但联合治疗组无统计学差异。

## 3 讨论

随着新药的出现及对症支持治疗手段的进步，标准治疗后的抗肿瘤治疗已经成为更多医生及患者的选择。但目前国外涉及四线治疗的研究极少，国内未见相关报道。我们的研究中，约8%的患者接受了探索性四线治疗，而国外报道为5%-20%，我们研究中的四线化疗比例较高，但与国外报道相似，可能与本研究纳入病例时间跨度较大，且近年来多数患者既往接受过靶向治疗有关^[2, 4, 5]^。我们的人群特点部分与日本研究类似，即患者的平均年龄低于其他研究，纳入了更多的非鳞癌及不吸烟患者。但我们的患者分期更晚，均为Ⅳ期，一般情况更差，四线治疗时ECOG评分高的患者高达50%。我们首次对*EGFR*突变情况进行了分析，而其他研究均没有报道相关结果^[2, 4-6]^。已有的几项研究结果有一定差异。Massarelli^[7]^的研究中四线治疗的PR为0，DCR为21%。而近期的两个研究中，可评价患者的PR分别为10%、20%^[2, 6]^。本研究中，虽然约有20%患者不能评价疗效，但12.9%的患者获得了PR，DCR为25.7%，提示部分患者的确能从四线治疗中获益。

我们的研究回顾了单中心10年的治疗结果，获得了31.0个月及10.1个月的中位总生存及四线治疗生存时间。OS类似或高于*EGFR*突变人群^[8, 9]^。其中约1/3的生存贡献来自于四线及以后的治疗。而多项关于二线治疗的Ⅲ期研究^[10-14]^显示二线治疗的中位OS约为5个月-9个月。本研究中四线中位PFS为2.6个月，与多项二线治疗的Ⅲ期研究PFS类似。因此四线治疗的生存时间值得重视。

既往的相关研究结果不一致，较早的研究^[7]^表明即使是一般情况较好的患者，接受三线或四线化疗的OS均为4个月，与观察人群无差异。一项澳大利亚12年的回顾研究^[5]^显示：5%的患者接受了四线治疗，相应的OS仅为5.4个月。但近年的研究与我们的结果类似，显示出较长的四线治疗生存。一项纳入65例样本使用培美曲塞进行的三、四线治疗研究^[6]^中，其生存时间分别达到了11个月及13个月，较前期研究有明显延长。2012年发表的日本研究^[2]^中，约20%的患者使用了吉非替尼，初次治疗的OS及四线治疗的OS分别为15.3个月及9.9个月。多数研究者认为，高效、低毒新药的临床应用为患者四线及之后的治疗提供了机会，患者生存得到了延长。在我们的研究中不吸烟腺癌患者比例较高，且有一定的基因突变比例，这类患者无论化疗还是靶向治疗均有相对较高的获益率。

在四线治疗的预后因素分析中，我们的研究显示未发现针对PFS的独立预后因素。但单因素及多因素分析均显示：不同的治疗方案是四线OS的独立预后因素。化疗组较靶向治疗组四线OS延长。这一结果提示靶向治疗药物的后期使用可能疗效有限。但本研究中使用的EGFR-TKI均为可逆性，不可逆的EGFR-TKI的作用及使用时序仍需进一步探讨。另一个四线OS的独立预后因素为是否接受后续治疗。四线治疗后有无后续治疗的四线中位OS有统计学差异，数值相差一倍以上。提示若患者可耐受且愿意接受更多线的治疗可能对其总生存延长有意义。这一结果为进一步临床研究提供了依据。

本研究中，约63%的患者接受了四线化疗，35%的患者接受了含培美曲塞方案。化疗的中位四线生存期达到了12个月左右，较小分子靶向药物更长。既往培美曲塞作为四线治疗的生存期达到了13个月^[6]^，较其他不含培美曲塞的研究有明显延长。有研究者^[15]^认为培美曲塞可能较其他化疗药物有更好的获益。本研究并未显示出含培美曲塞方案的优越性。双药及单药化疗方案也无差异。我们的前期研究提示，二次使用EGFR-TKI药物仍有部分获益，但与基因状态及用药顺序有关^[16]^。本研究中仅有29%的患者证实*EGFR*基因突变，且为治疗前的*EGFR*基因状态。已有研究显示，经过化疗的*EGFR*基因状态可能发生改变^[17]^。因此，应在临床上推广病程中的多次活检及相关基因检测。另外，不可逆EGFR-TKI的出现可能为四线治疗提供更多的选择。四线治疗中EGFR-TKI的作用仍需进一步研究。

我们的研究结果显示：结合患者意愿进行的四线治疗，PR为12.9%，DCR为25.7%，四线治疗中位PFS为2.6个月，中位四线生存时间为10.1个月。化疗的中位四线生存期达到了11.7个月，患者有生存获益，其作用及地位值得进一步研究及讨论，但此类治疗的进行，需准确评估患者的一般情况及耐受性，比较可能的获益及风险。必须注意的是本研究为回顾性，偏倚不可避免，晚期非小细胞肺癌四线治疗的作用需要前瞻性临床试验进一步探讨。

## References

[b1] 1NCCN Clinical Practice Guidelines in Oncology (NCCN Guidelines). Non-small cell lung cancer. National Comprehensive Cancer Network, 2014.

[2] Asahina H, Sekine I, Horinouchi H (2012). Retrospective analysis of third-line and fourth-line chemotherapy for advanced non-small-cell lung cancer. Clin Lung Cancer.

[3] Matsuyama R, Reddy S, Smith TJ (2006). Why do patients choose chemotherapy near the end of life? A review of the perspective of those facing death from cancer. J Clin Oncol.

[4] Murillo JR, Koeller J (2006). Chemotherapy given near the end of life by community oncologists for advanced non-small cell lung cancer. Oncologist.

[5] Fiegl M, Hilbe W, Auberger J (2008). Twelve-year retrospective analysis of lung cancer--The TYROL Study: Daily routine in 1, 424 patients (1995-2006). J Clin Oncol.

[6] Chang MH, Ahn JS, Lee J (2010). The efficacy of pemetrexed as a third-or fourth-line therapy and the significance of thymidylate synthase expression in patients with advanced non-small cell lung cancer. Lung Cancer.

[7] Massarelli E, Andre F, Liu DD (2003). A retrospective analysis of the outcome of patients who have received two prior chemotherapy regimens including platinum and docetaxel for recurrent non-small-cell lung cancer. Lung Cancer.

[8] Maemondo M, Inoue A, Kobayashi K (2010). Gefitinib or chemotherapy for non-small-cell lung cancer with mutated *EGFR*. N Engl J Med.

[9] Mitsudomi T, Morita S, Yatabe Y (2009). Gefitinib versus cisplatin plus docetaxel in patients with non-small-cell lung cancer harbouring mutations of the epidermal growth factor receptor (WJTOG3405): An open label, randomised phase 3 trial. Lancet Oncol.

[10] Fossella FV, DeVore R, Kerr RN (2000). Randomized phase Ⅲ trial of docetaxel versus vinorelbine or ifosfamide in patients with advanced non-small-cell lung cancer previously treated with platinum-containing chemotherapy regimens. J Clin Oncol.

[11] Hanna N, Shepherd FA, Fossella FV (2004). Randomized phase Ⅲ trial of pemetrexed versus docetaxel in patients with non-small-cell lung cancer previously treated with chemotherapy. J Clin Oncol.

[12] Kim ES, Hirsh V, Mok T (2008). Gefitinib versus docetaxel in previously treated non-small-cell lung cancer (INTEREST): a randomised phase Ⅲ trial. Lancet.

[13] Thatcher N, Chang A, Parikh P (2005). Gefitinib plus best supportive care in previously treated patients with refractory advanced non-small-cell lung cancer: results from a randomised, placebo-controlled, multicentre study (Iressa Survival Evaluation in Lung Cancer). Lancet.

[14] Shepherd FA, Rodrigues PJ, Ciuleanu T (2005). Erlotinib in previously treated non-small-cell lung cancer. N Engl J Med.

[15] Syrigos KN, Saif MW, Karapanagiotou EM (2011). The need for third-line treatment in non-small cell lung cancer: an overview of new options. Anticancer Res.

[16] Luo DX, Huang MJ, Zhang XX (2012). Salvage treatment with erlotinib after gefitinib failure in advanced non-small-cell lung cancer patients with poor performance status: A matched-pair case-control study. Thoracic Cancer.

[17] Bai H, Wang Z, Chen K (2012). Influence of chemotherapy on *EGFR* mutation status among patients with non-small-cell lung cancer. J Clin Oncol.

